# Supplementary material for: OpenMM 7: Rapid development of high performance algorithms for molecular dynamics
Source: PLoS Comput Biol. 2017 Jul 26;13(7):e1005659. doi: 10.1371/journal.pcbi.1005659 (PMC5549999; doi:10.1371/journal.pcbi.1005659)
Supplement: S1 Source Code — (ZIP) [file pcbi.1005659.s002.zip › OpenMM-7.0.1-Source/docs/api-c++/generated/OpenMM.DrudeLangevinIntegrator.html]

DrudeLangevinIntegrator — OpenMM @OPENMM\_MAJOR\_VERSION@.@OPENMM\_MINOR\_VERSION@ documentation


# DrudeLangevinIntegrator¶

*class* `OpenMM::``DrudeLangevinIntegrator`¶
:   This `Integrator` simulates systems that include Drude particles. It applies two different Langevin thermostats to different parts of the system. The first is applied to ordinary particles (ones that are not part of a Drude particle pair), as well as to the center of mass of each Drude particle pair. A second thermostat, typically with a much lower temperature, is applied to the relative internal displacement of each pair.

    This integrator can optionally set an upper limit on how far any Drude particle is ever allowed to get from its parent particle. This can sometimes help to improve stability. The limit is enforced with a hard wall constraint.

    This `Integrator` requires the `System` to include a `DrudeForce`, which it uses to identify the Drude particles.

    Methods

    |  |  |
    | --- | --- |
    | `DrudeLangevinIntegrator` | Create a `DrudeLangevinIntegrator`. |
    | `getTemperature` | Get the temperature of the main heat bath (in Kelvin). |
    | `setTemperature` | Set the temperature of the main heat bath (in Kelvin). |
    | `getFriction` | Get the friction coefficient which determines how strongly the system is coupled to the main heat bath (in inverse ps). |
    | `setFriction` | Set the friction coefficient which determines how strongly the system is coupled to the main heat bath (in inverse ps). |
    | `getDrudeTemperature` | Get the temperature of the heat bath applied to internal coordinates of Drude particles (in Kelvin). |
    | `setDrudeTemperature` | Set the temperature of the heat bath applied to internal coordinates of Drude particles (in Kelvin). |
    | `getDrudeFriction` | Get the friction coefficient which determines how strongly the internal coordinates of Drude particles are coupled to the heat bath (in inverse ps). |
    | `setDrudeFriction` | Set the friction coefficient which determines how strongly the internal coordinates of Drude particles are coupled to the heat bath (in inverse ps). |
    | `getMaxDrudeDistance` | Get the maximum distance a Drude particle can ever move from its parent particle, measured in nm. |
    | `setMaxDrudeDistance` | Set the maximum distance a Drude particle can ever move from its parent particle, measured in nm. |
    | `getRandomNumberSeed` | Get the random number seed. |
    | `setRandomNumberSeed` | Set the random number seed. |
    | `step` | Advance a simulation through time by taking a series of time steps. |

    `DrudeLangevinIntegrator`(double *temperature*, double *frictionCoeff*, double *drudeTemperature*, double *drudeFrictionCoeff*, double *stepSize*)¶
    :   Create a `DrudeLangevinIntegrator`.

        |  |  |
        | --- | --- |
        | Parameters: |  |

        - **temperature** – the temperature of the main heat bath (in Kelvin)
        - **frictionCoeff** – the friction coefficient which couples the system to the main heat bath (in inverse picoseconds)
        - **drudeTemperature** – the temperature of the heat bath applied to internal coordinates of Drude particles (in Kelvin)
        - **drudeFrictionCoeff** – the friction coefficient which couples the system to the heat bath applied to internal coordinates of Drude particles (in inverse picoseconds)
        - **stepSize** – the step size with which to integrator the system (in picoseconds)

    double `getTemperature`() *const*¶
    :   Get the temperature of the main heat bath (in Kelvin).

        |  |  |
        | --- | --- |
        | Returns: | the temperature of the heat bath, measured in Kelvin |

    void `setTemperature`(double *temp*)¶
    :   Set the temperature of the main heat bath (in Kelvin).

        |  |  |
        | --- | --- |
        | Parameters: |  |

        - **temp** – the temperature of the heat bath, measured in Kelvin

    double `getFriction`() *const*¶
    :   Get the friction coefficient which determines how strongly the system is coupled to the main heat bath (in inverse ps).

        |  |  |
        | --- | --- |
        | Returns: | the friction coefficient, measured in 1/ps |

    void `setFriction`(double *coeff*)¶
    :   Set the friction coefficient which determines how strongly the system is coupled to the main heat bath (in inverse ps).

        |  |  |
        | --- | --- |
        | Parameters: |  |

        - **coeff** – the friction coefficient, measured in 1/ps

    double `getDrudeTemperature`() *const*¶
    :   Get the temperature of the heat bath applied to internal coordinates of Drude particles (in Kelvin).

        |  |  |
        | --- | --- |
        | Returns: | the temperature of the heat bath, measured in Kelvin |

    void `setDrudeTemperature`(double *temp*)¶
    :   Set the temperature of the heat bath applied to internal coordinates of Drude particles (in Kelvin).

        |  |  |
        | --- | --- |
        | Parameters: |  |

        - **temp** – the temperature of the heat bath, measured in Kelvin

    double `getDrudeFriction`() *const*¶
    :   Get the friction coefficient which determines how strongly the internal coordinates of Drude particles are coupled to the heat bath (in inverse ps).

        |  |  |
        | --- | --- |
        | Returns: | the friction coefficient, measured in 1/ps |

    void `setDrudeFriction`(double *coeff*)¶
    :   Set the friction coefficient which determines how strongly the internal coordinates of Drude particles are coupled to the heat bath (in inverse ps).

        |  |  |
        | --- | --- |
        | Parameters: |  |

        - **coeff** – the friction coefficient, measured in 1/ps

    double `getMaxDrudeDistance`() *const*¶
    :   Get the maximum distance a Drude particle can ever move from its parent particle, measured in nm. This is implemented with a hard wall constraint. If this distance is set to 0 (the default), the hard wall constraint is omitted.

    void `setMaxDrudeDistance`(double *distance*)¶
    :   Set the maximum distance a Drude particle can ever move from its parent particle, measured in nm. This is implemented with a hard wall constraint. If this distance is set to 0 (the default), the hard wall constraint is omitted.

    int `getRandomNumberSeed`() *const*¶
    :   Get the random number seed. See `setRandomNumberSeed()` for details.

    void `setRandomNumberSeed`(int *seed*)¶
    :   Set the random number seed. The precise meaning of this parameter is undefined, and is left up to each `Platform` to interpret in an appropriate way. It is guaranteed that if two simulations are run with different random number seeds, the sequence of random forces will be different. On the other hand, no guarantees are made about the behavior of simulations that use the same seed. In particular, Platforms are permitted to use non-deterministic algorithms which produce different results on successive runs, even if those runs were initialized identically.

        If seed is set to 0 (which is the default value assigned), a unique seed is chosen when a `Context` is created from this `Force`. This is done to ensure that each `Context` receives unique random seeds without you needing to set them explicitly.

    void `step`(int *steps*)¶
    :   Advance a simulation through time by taking a series of time steps.

        |  |  |
        | --- | --- |
        | Parameters: |  |

        - **steps** – the number of time steps to take

High performance molecular simulation on GPUs


### Navigation

- Core Objects
- Forces
- Integrators
- Extras

©2015, Stanford University and the Authors.
|
Powered by Sphinx 1.3.1
& Alabaster 0.7.7
|
Page source
